# LifeWatch ERIC: papers collection on original datasets and new e-services for the biodiversity and ecosystems’ scientific community

**DOI:** 10.3897/BDJ.12.e119804

**Published:** 2024-02-08

**Authors:** Christos Arvanitidis, Alberto Basset, Peter van Tienderen, Lucas de Moncuit, Cristina Isabel Huertas Olivares, Cristina Di Muri, Ana Mellado, Wouter Los

**Affiliations:** 1 LifeWatch ERIC, Seville, Spain LifeWatch ERIC Seville Spain; 2 Institute of Marine Biology, Biotechnology and Aquaculture, Hellenic Centre for Marine Research, Herakleion, Greece Institute of Marine Biology, Biotechnology and Aquaculture, Hellenic Centre for Marine Research Herakleion Greece; 3 University of Salento, Lecce, Italy University of Salento Lecce Italy; 4 LifeWatch ERIC, Lecce, Italy LifeWatch ERIC Lecce Italy; 5 University of Amsterdam, Amsterdam, Netherlands University of Amsterdam Amsterdam Netherlands; 6 National Research Council (CNR), Research Institute on Terrestrial Ecosystems (IRET), Lecce, Italy National Research Council (CNR), Research Institute on Terrestrial Ecosystems (IRET) Lecce Italy

**Keywords:** LifeWatch ERIC, biodiversity, ecology, Research Infrastructure, e-Science Infrastructure

## Abstract

Papers including articles that are produced because of the activities of LifeWatch ERIC, in the context of its second implementation period (2022 - 2026) and through the implementation of its new Strategic Working Plan, are published in this special collection. The articles include data papers, papers describing the development and functioning of analytical services and papers describing any other research outcome, produced either by LifeWatch ERIC or by any collaboration with any other ERIC, Research Infrastructure, global aggregator or other legal entity.

## Introduction

LifeWatch ERIC is a distributed Research Infrastructure Consortium on biodiversity, from genes to ecosystems, composed of eight European Union Member States: Belgium, Bulgaria, Greece, Italy, the Netherlands, Portugal, Slovenia and Spain.

LifeWatch ERIC provides e-Science research facilities and services to scientists investigating biodiversity and ecosystems towards addressing key societal challenges, such as those linked to climate change, resource efficiency, food security and agriculture, sustainable development, energy and health.

LifeWatch ERIC’s members operate from national nodes, known as Distributed Centres, while its Common Facilities are located in three Member States: Spain (Statutory Seat Office and ICT-Core), Italy (Service Centre) and the Netherlands (Virtual Laboratories and Innovations Centre).

After a preparatory phase (2006 – 2010) and a transition phase (2010 – 2017), LifeWatch was established as a European Research Infrastructure Consortium (ERIC) by the European Commission, entering its operational phase, with its first implementation period covering the years 2017 – 2021. In 2022, LifeWatch ERIC (LW ERIC) begins its second five-year implementation period.

## Vision

The long-term vision behind LifeWatch ERIC is to become the Research Infrastructure providing access to the world's biodiversity content, services and communities with one click.

## Mission

During its second implementation period, LifeWatch ERIC aims to accelerate the research efforts of the scientific community by delivering a European state-of-the-art e-Science Research Infrastructure on biodiversity and ecosystem research: a Digital Twin which: (a) provides access to, and support for, key scientific services by applying cutting-edge ICT technology, (b) enables reproducible analytics, (c) is co-designed and co-created with the user communities and (d) is tuned with the needs for research that provides key insights for society, in particular science-based policy.

## Culture Principles

**Figure 1.**
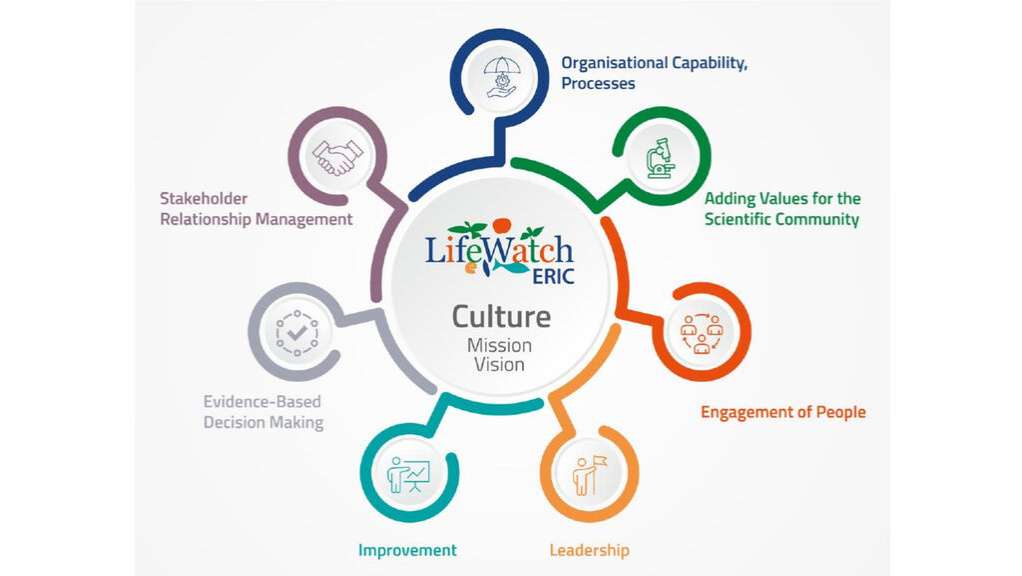
LifeWatch Research schematic representation of its Culture Principles.

The LW ERIC Culture Principles are the building blocks of its collective DNA and the pillars of its organisational personality (see Fig. 1).

They also serve as the foundation for creating the positive socio-economic impacts which ERICs are meant to enable, so that staff, researchers and all partners can be proud to work with LW ERIC. The seven Culture Principles have been endorsed by the Executive Board, who invite employees and partners to contribute to the culture development effort by applying these principles in every aspect of their work.

## Strategic objectives (SO)

(SO1): To industrialise and support the knowledge and technology transfer mechanisms of the existing prototype LW ERIC Research Infrastructure at all levels: scientific, technical, communication, innovation, administrative and financial, from current Technology Readiness Level 6 to 9.

(SO2): To consolidate and broaden the LW ERIC e-Science Infrastructure towards integration of all content, services and other resources (e.g. installations, hardware, software, observatories), currently existing in the Member States, as well as new ones, into a single Research Infrastructure offering an open, creative and democratic space to its users. Its unique Scientific Knowledge Graph (SKG) System will perform this consolidation.

(SO3): To advance scientific and technological innovation, based on the continuous improvement in the performance of the Virtual Research Environment (VRE) by investing in emerging technologies with profound application in Biodiversity and Ecosystem Research (BER) (e.g. Artificial Intelligence-Machine-Learning, smart HPC-Cloud-Edge Computing and Blockchain technologies), towards the next generation Infrastructure on Biodiversity and Ecosystem Research (next-gen IBER).

(SO4): To deepen the engagement of the scientific communities (with attention to diversity, inclusivity and equity), stakeholders and citizens working on biodiversity and ecological observatories, at a global scale.

(SO5): To forge collaboration with the public sector, private sector and industry to guarantee the take-up and sustainability of the innovation produced and to address aspects of the EU Green Deal, EU Biodiversity 2030 and EU Digitisation and Innovation plans.

## Key actions for scientific communities and stakeholders

(a) Membership expansion activities along with a strategy to increase membership and liaise with other (including non-European) countries/organisations. They originate from the LW ERIC Document on the Strategy to Expand Membership, along with the main targets to be contacted during the next implementation period.

(b) Investing in the trading zones commonly identified with other ERICs, Research Infrastructure and global aggregators, for the development of the synthetic science and innovation.

(c) Engaging the scientific communities and stakeholders, as well as individual researchers and engineers (developers), mapping their research needs and co-design and co-develop their identified strategic services.

(d) Liaising activities with strategic communities and biodiversity/ecological international societies (e.g. EEF, EMBS), networks (e.g. MBON, MARS/WAMS, ALTERnet, UNECE-CLRTAP, ETC Biological Diversity-EIONET etc.) and observatories.

(e) Liaising activities with strategic networks of citizen scientists.

## Preparatory Phase

**Figure 2.**
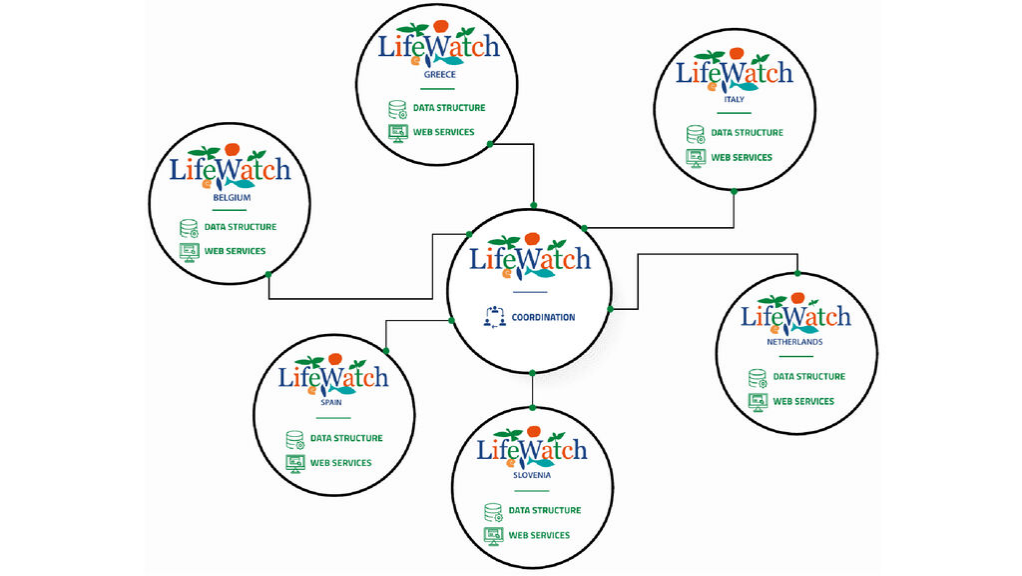
LifeWatch Research Infrastructure during its first years of development (before it became an ERIC).

During its preparatory phase, LifeWatch was designed as a completely new and ambitious Research Infrastructure (Fig. 2), opening an innovative approach to scientific research in the field of biodiversity and ecosystems by integrating biological and ecological sciences and technologies, on the one hand, with information and communication sciences and technologies on the other.

## Transition Phase

In the subsequent phase, LifeWatch developed as a loosely connected ecosystem of nodes, which grew their content and services structure depending on the needs of their scientific communities (Fig. 2). During that first period of development, the three Member States which took the responsibility of hosting the LifeWatch Common Facilities, acted as coordinating hubs in the network of national nodes and prepared the request to become an ERIC.

## First Implementation Period

**Figure 3.**
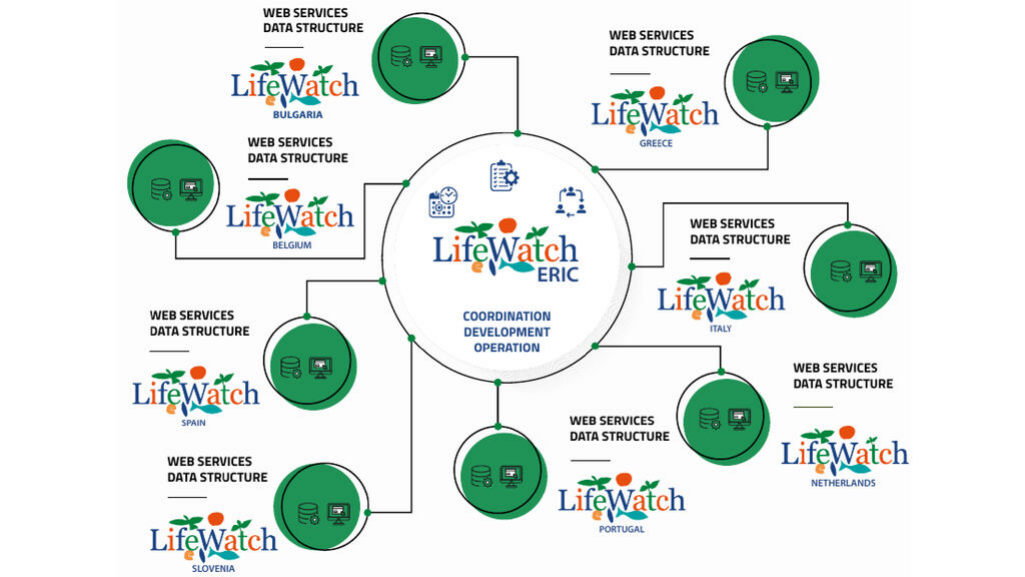
LifeWatch Research Infrastructure during its first five years of implementation as an ERIC.

During the first 5-year implementation period, LW ERIC transformed into a real Research Infrastructure with its Common Facilities and Distributed Centres (Fig. 3).

The essential ingredients for LW ERIC are: (1) coordination and management of the trustworthy, federated distributed e-Science Research Infrastructure; (2) open-access (FAIR: Findable, Accessible, Interoperable, Reusable) data; (3) reproducible analytics; (4) mobilised communities. The main challenges identified during this first implementation period were of a scientific, technical and cultural nature, the last type being the most difficult to tackle:

"*to change scientists' everyday habits, by opening the LifeWatch Research Infrastructure webpage as they turn on their PCs to use their preferred Virtual Research Environments. This change would direct most of the scientific effort from a single-core brain operation or “brain-etics”, into high-performance brain network synthesis or “brain-omics”. This is a cultural change we have to push forward in our community*".

By pursuing these ingredients and especially through its flagship project, the Internal Joint Initiative, LW ERIC created a prototype of its infrastructure in terms of human capital, organisation, services and Virtual Research Environments, integration and community engagement. While starting and further boosting construction, LW ERIC was already operational.

The essential elements of the prototype are: Catalogue of Resources (Metadata Catalogue); Repository of Semantic Resources (e.g. Controlled Vocabularies, Thesauri, and Ontologies: EcoPortal); FAIR compliant datasets (1,502 in total); Web services (113); Thematic services (11); Virtual Research Environments (12); Workflows (5); Training resources (25); Research sites (10); LifeBlock (first Research Infrastructure applied BlockChain technology); Tesseract and its Virtual Research Environments building platform (including Jupyter Notebook) (technical composability layer); Network of communities engaged (numbers refer to 1 January 2023 and are continuously growing).

## Second Implementation Period: From prototype to Research Infrastructure

The next 5-year implementation period (2022 - 2026) will witness LW ERIC becoming a fully operational Research Infrastructure, compliant with the ESFRI criteria for a Landmark ERIC. It will bring LW ERIC to a stage of continuous acceleration by industrialising its prototype in order to consolidate the resources developed either by the Distributed Centres or the Common Facilities. It will undergo continuous upgrading and (co-)construction, responding to the needs of its target communities and stakeholders. This industrialisation process, undertaken together with the engaged communities and stakeholders, is essential to assemble all independent pieces contributed by the Member Countries and to enable collaborative development through interoperability. The independent data, software, publications and other types of research products will be assembled into systems. The above will push the Technology Readiness Level of the LW ERIC prototype from 6 to 9.

## Second Implementation Period: The layers of the Research Infrastructure

**Figure 4.**
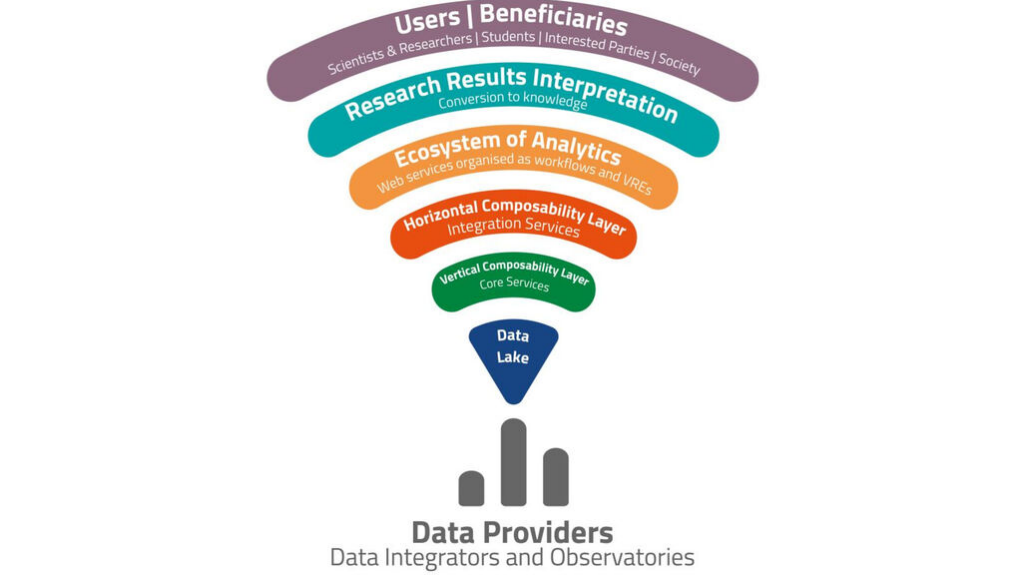
Design of the Research Infrastructure to be delivered during the next implementation period of LW ERIC.

An oversimplified conceptual representation of the main layers of this prototype evolving into a Research Infrastructure is depicted in Fig. 4.

The basis of the Infrastructure is formed by the data providers, data integrators and data observatories, all contributing data to the *Data Lake* of LW ERIC. On top of this Data Lake, *four layers of services* will be developed which follow the entire scientific research life cycle from hypothesis and data collection to interpretation of results and the publication of new knowledge, which inspires the motto of the next-generation Infrastructure on Biodiversity and Ecosystem Research: "*from measurement to manuscript*".

The *first layer* includes the *core services* of the LW ERIC infrastructure (e.g. AAI, HPC, cloud services, storage, personal space). These services enable the "*vertical composability*" of all the content and services uploaded and operated by the Infrastructure, that is, their connection with the previously mentioned core features of the Infrastructure so that they become functional through the next-generation Infrastructure on Biodiversity and Ecosystem Research.

The *second layer* is formed by the *integration services*. These include examples of the disruptive technologies which were applied during the previous period, such as the Tesseract and its Virtual Research Environment building platform (including Jupyter Notebook), LifeBlock, Metadata Catalogue and EcoPortal. Their function is the "*horizontal composability*" of the content, services and any other research product, that is, the connection to each other so that they can be used in any possible combination. This combination can even be made *on-the-fly* by users, subject to the demands of their research.

The *third layer* includes the *ecosystem of analytics*, which is composed of web services (1), to be used either individually or in combination in the form of organised workflows (2) and Virtual Research Environments (3) (listed below).

The *fourth layer* is composed by services which accommodate the needs for *converting the research results into knowledge* (e.g. mapping services, summary statistics, ichnographics, publication services). At this top layer, there are several types of *users* benefitting from the Infrastructure.

In the new implementation period, an important investment in disruptive technologies (e.g. AI) will be attempted. Their application will be exemplary in accelerating the value and multiplying the use of the current resources (e.g. content, services) of LW ERIC. Definitely, the participation of LW ERIC in ongoing Horizon Europe projects, such as BiCIKL, EOSC Future, FAIR-IMPACT, Biodiversity Digital Twin and AroServ, as well as the common investments with the key-players (ERICs, RIs), will be instrumental in achieving such a successful investment.

1. The definition of Wikipedia is adopted here for a web service (https://en.wikipedia.org/wiki/Web_service): A web service is either: (a) a service offered by an electronic device to another electronic device, communicating with each other via the Internet or (b) a server running on a computer device, listening for requests at a particular port over a network, serving web documents (HTML, JSON, XML, images).
2. The definition of Wikipedia is adopted here for a workflow (https://en.wikipedia.org/wiki/Workflow): A workflow consists of an orchestrated and repeatable pattern of activity, enabled by the systematic organisation of resources into processes that transform materials, provide services or process information. It can be depicted as a sequence of operations, the work of a person or group, the work of an organisation of staff or one or more simple or complex mechanisms.
3. The definition of Wikipedia is adopted here for a virtual research environment (https://en.wikipedia.org/wiki/Virtual_research_environment): A Virtual Research Environment (VRE) (or Virtual Laboratory) is an online system helping researchers collaborate. Features usually include collaboration support (Web forums and wikis), document hosting and some discipline-specific tools, such as data analysis, vizualisation or simulation management. In some instances, publication management and teaching tools, such as presentations and slides, may be included. VREs have become important in fields where research is primarily carried out in teams which span institutions and even countries: the ability to easily share information and research results is valuable.

NB: The European Open Science Cloud (EOSC) does not yet include such definitions.

## Advantages over other ERICs and Research Infrastructure

Right from the start of its first implementation period, LW ERIC was designed to be a *systemic solution*, which means it unites all the separate components developed at national level. As a result, each component benefits from the attributes of the technologies involved and each becomes part of the broader LW ERIC Infrastructure.

This *systemic, holistic approach* is radically different from the *piecemeal approach* followed by several key players in the landscape. Thus far, they have been delivering one piece of software at a time, depending on the specific problems and needs of the communities they serve. This practice has led to a *patchwork solution*, with infrastructure components not organically linked to each other and, therefore, incompatible with many types of workflows, thematic services, Virtual Research Environment etc. other than the web services themselves. Typically, the components in many Research Infrastructure are provided to the user one at a time, that is, the user can use only one component, then the next and so on. Additionally, each of these components has its own specifications and application rules.

LifeWatch ERIC brings an *innovative solution* to the above issue: its *integration services* (e.g. Tesseract and its Virtual Research Environment building platform (including Jupyter Notebook), LifeBlock (SKG System), Metadata Catalogue and EcoPortal) are capable of connecting and integrating both the content and services developed by the *national operators*. This means that both *content and services* can be *used individually*, but also *in combination*, with the output of one service serving as an input for the next and so on.

An important advantage of this solution is that the resources created by the investments of Member States as part of the LW ERIC Infrastructure are continuously being upgraded and, therefore, their *value increases with time*. The option to combine multiple components has the potential to *attract more users* than they currently do.

An additional advantage of the LW ERIC Infrastructure is that the services (software) it develops are *discipline- and domain-agnostic* and, therefore, may be used by multiple disciplines and domains. A good example is the micro-CT vLab developed by LifeWatchGreece which has been used so far by scientists working not only in biodiversity and ecosystems, but also in material science, veterinary science and cardiology. This means that both the *content and services* developed by LW ERIC *can benefit additional users* from *multiple disciplines and domains*. This wider accessibility clearly provides added value to the content and services developed at the national scale.

## Unique attributes

By following the *holistic approach*, the LW ERIC Infrastructure offers *unique attributes*, such as:

- Providing access to *multiple data* and *service resources* with *one click*, for example, biological and environmental data at the same time;
- Providing *workflows* dedicated to specific lines of research with *customised data* and *services*;
- Enabling *users to develop* their *own workflows on-the-fly*, depending on the working hypotheses and the content and services available;
- *Serving* researchers from *multiple disciplines/domains* by providing *multiple workflows* that can test the same hypothesis in parallel and then compare the results.

## Implications

The above attributes have already brought important *implications to the way scientific research is being pursued*:

- A *holistic approach* which encompasses all stages in the life cycle of scientific research practice, that is, from *hypothesis formation* to *publication of new knowledge*;
- *The application and further development of disruptive technology which takes the transparency and repeatability* of doing research to a much higher level than it is currently at;
- Promotion of *multi-disciplinary* and *cross-domain* research, which brings down the existing barriers between them and encourages the communities to work on the same collaborative environment;
- Collaboration with the EOSC next-generation e-Infrastructure.

All of the above contribute to *synthetic knowledge*, that is, *testing* the *same hypothesis* with *content and services* from a *plethora* of *disciplines/domains* and *comparing* the *results* to *achieve* a *knowledge basis much broader than it is today*. This way, the new scientific knowledge will have a much *stronger convincing power*.

The above implications also assist the current ERICs, Research Infrastructures and international organisations to find their *trading zones* and to invest together with them to further scientific and technological innovation. This approach will continue from the mapping exercise already in progress with many of the ERICs and RIs towards the development of common and inter-dependent resources (hardware, data, services, common projects etc.).

Finally, these attributes bring another, equally important, implication for LW ERIC: they allow the Infrastructure to act as a *marketplace* for both *content and services* on biodiversity and ecosystem research. This will have the *potential* to attract major actors in the public sector, private sector and industry to join and invest together with LW ERIC.

